# Evaluation of left ventricular outflow tract obstruction with 4D phase contrast in patients with hypertrophic cardiomyopathy

**DOI:** 10.1186/1532-429X-16-S1-P312

**Published:** 2014-01-16

**Authors:** Linda Chu, Celia P Corona-Villalobos, Mehmet A Gulsun, Steven Shea, Michael Markl, Theodore Abraham, David Bluemke, Ihab R Kamel, Stefan L Zimmerman

**Affiliations:** 1 Johns Hopkins University, Baltimore, Maryland, USA; 2 Siemens Corporate Research, Baltimore, Maryland, USA; 3 Northwestern University, Chicago, Illinois, USA; 4 National Institute of Health, Bethesda, Maryland, USA

## Background

Patients with hypertrophic cardiomyopathy (HCM) develop left ventricular outflow tract (LVOT) obstruction due to combination of asymmetric septal hypertrophy and systolic anterior motion of the mitral leaflet. Degree of LVOT obstruction is an important predictor in patient symptoms and clinical outcome. LVOT obstruction is currently assessed by Doppler echocardiography (echo) using peak velocity to calculate pressure gradient in the LVOT. 4D phase contrast (4D PC) MRI is an emerging technique for quantification of regional flow and velocity. 4D PC MRI is a 3 dimensional, 3 directionally encoded, time resolved (cine) velocity acquisition. It has the advantage of providing full 3D volume coverage and flexibility of retrospective analysis of flow at any location and in any imaging plane. In this preliminary study, we evaluated LVOT obstruction in HCM patients with 4D PC technique, using echo as reference standard.

## Methods

In this IRB-approved prospective study, patients who presented to cardiac MRI for evaluation of HCM from July 2012 to June 2013 were recruited to undergo 4D PC protocol in addition to standard cardiac MRI protocol. 4D PC sequence was acquired during free-breathing, using a navigator-gated gradient-echo pulse-sequence with interleaved 3D flow-encoding and prospective ECG-gating. 4D PC data was post-processed on separate workstation using Siemens 4D Flow investigational prototype [1] by particle seeding and generation of pathlines to depict blood flow through left ventricle. Post-processed pathline cine data were reviewed by two blinded reviewers to determine presence or absence of LVOT obstruction. LVOT obstruction was compared with echo obstruction classification and LVOT pressure gradient using kappa statistic and student's t-test.

## Results

23 patients (mean age 52.2 years, 18 males, 5 females) completed the 4D PC MRI. 17 patients were confirmed to have HCM on clinical evaluation. Remaining 6 patients did not have HCM or were undergoing additional workup to exclude HCM. 4D PC qualitative assessment showed non-obstructive pattern in 12 patients and obstructive pattern in 11 patients. 20 of the 23 patients had corresponding echo data, with 10 non-obstructive, 3 labile obstructive, and 7 obstructive patterns. Kappa statistic comparing presence and absence of LVOT obstruction for 4D PC and echo was 0.700 (p = 0.002). There was significant different in LVOT pressure gradients for non-obstructive vs. obstructive groups, with p = 0.008 for resting gradient and p < 0.001 for peak provoked gradient.

## Conclusions

4D PC MRI is an emerging MRI technique that can be used to assess for LVOT obstruction in HCM patients.

## Funding

Linda Chu received research support from RSNA R&E Foundation Fellow Grant.

**Figure 1:**
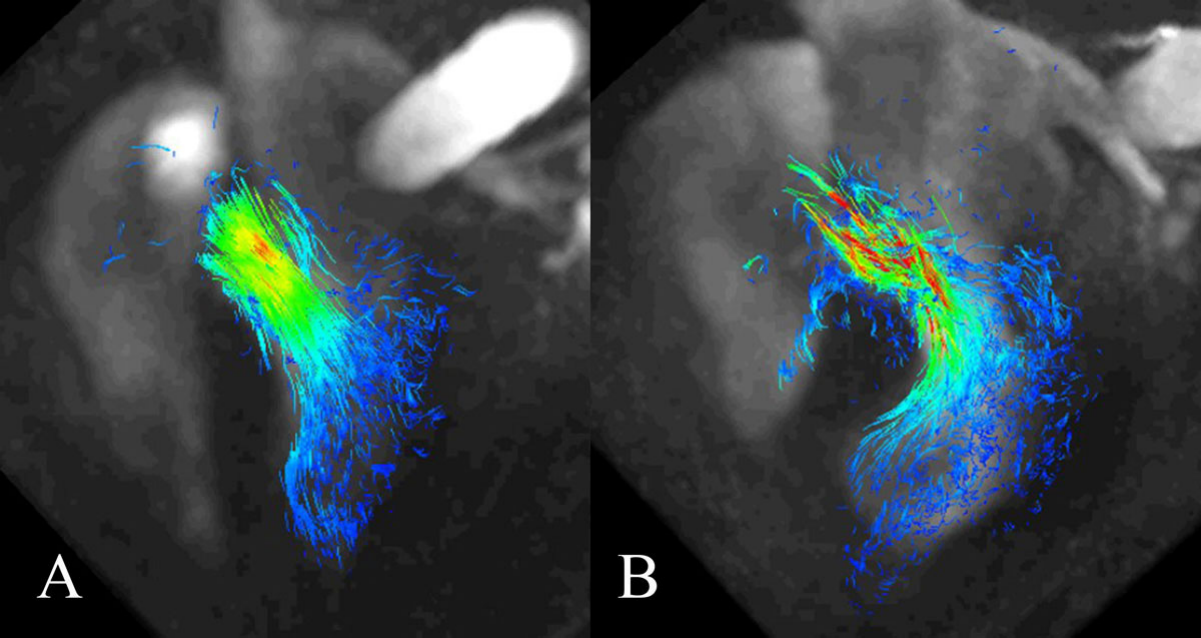
**4D phase contrast pathline visualization of blood flow along LVOT**. A) Pathline visualization of nonobstructive patient shows laminar flow in the LVOT. B) Pathline visualization of severely obstructive patient shows disordered and turbulent flow in the LVOT.

**Figure 2:**
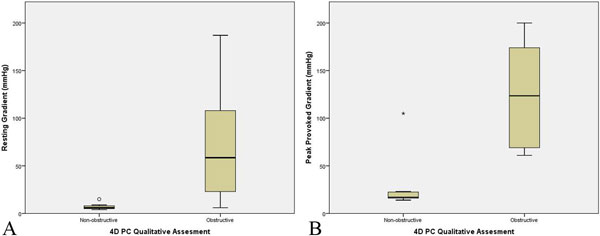
**Boxplots of A) resting gradient and B) peak provoked gradient along LVOT vs. 4D PC qualitative assessment of LVOT obstruction show significant different in resting and peak provoked pressure gradients between non-obstructive and obstructive groups**.
